# "The metabolic syndrome... is dead": These reports are an exaggeration

**DOI:** 10.1186/1475-2840-10-11

**Published:** 2011-01-27

**Authors:** Alexander Tenenbaum, Enrique Z Fisman

**Affiliations:** 1 Sackler Faculty of Medicine, Tel-Aviv University, 69978 Tel-Aviv, Israel; 2 Cardiovascular Diabetology Research Foundation, 58484 Holon, Israel; 3 Cardiac Rehabilitation Institute, Sheba Medical Center, 52621 Tel-Hashomer, Israel

## Abstract

The debates continue over the validity of the metabolic syndrome concept. The continuous increment of the obesity pandemic is almost worldwide paralleled by rising rates of metabolic syndrome prevalence. Then, it seems obvious that these debates drove the need for further investigations as well as a deeper cooperation between relevant national and international organizations regarding the issue. Instead, part of the scientific community elected to totally "dismiss" the concept of the metabolic syndrome. Meanwhile, *the best available evidence *from three consecutive large meta-analyses has systematically shown that people with metabolic syndrome are at increased risk of cardiovascular events. The most recent and largest of them included near one million patients (total n = 951,083). The investigators concluded that the metabolic syndrome is associated with a 2-fold increase in cardiovascular outcomes and a 1.5-fold increase in all-cause mortality rates. One of the ways to hit the metabolic syndrome is an utterly simplistic view on this concept as a predictive tool only. Of course, the presence of the metabolic syndrome possesses a definite predictive value, but first of all it is a widely accepted concept regarding a biological condition based on the complex and interrelated pathophysiological mechanisms starting from excess central adiposity and insulin resistance. Therefore, it is completely unfair to compare it with statistically constructed predictive tools, including stronger prognostic variables even unrelated to each other from the biological point of view. For example, in the criteria for metabolic syndrome (in contrast to Framingham score) age and cholesterol - presumably low density lipoprotein - cholesterol (LDL-C) - levels are not included, as well as a variety of strong predictors used in other risk-stratification scores: previous myocardial infarction, heart failure, smoking, family history, etc. However, the metabolic syndrome identifies additional important residual vascular risk mainly associated with insulin resistance and atherogenic dyslipidemia (low high density lipoprotein-cholesterol (HDL-C), high triglycerides, small, dense LDL-C). Therefore, the metabolic syndrome could be a useful additional contributor in estimation of global cardiovascular risk beyond age, high LDL-C or other standard risk factors. The components of the metabolic syndrome have partially *overlapping *mechanisms of pathogenic actions mediated through common metabolic pathways. Therefore their total combined effect could be less than the summed of the individual effects. The concept that the metabolic syndrome is a consequence of obesity and insulin resistance, provides a useful "life-style changes" approach for prevention and treatment: caloric restriction, weight-loss and increased physical activity. The next step could theoretically be pharmacological interventions such as metformin, acarbose, fibrates, weight-loss drugs (currently only orlistat is practically available) and perhaps glucagon-like peptide-1 agonists. A third step should probably be kept for bariatric surgery.

## 

The diagnostic criteria for the metabolic syndrome are not ideal. Controversy continues over the validity of its naming, as well as disagreement over its relevance as a practical clinical tool. One of the important questions which still remain open is its predictive value: does the metabolic syndrome forecast cardiovascular events, diabetes or disease progression any better than the sum of its components?

The continuous increment of the obesity pandemic is almost worldwide paralleled by rising rates of metabolic syndrome prevalence. Then, it seems obvious that these debates drove the need for further investigations as well as a deeper cooperation between relevant national and international organizations aiming to improve and unify the terms [1,2]. Surprisingly, part of the scientific community elected to totally "dismiss" the concept of the metabolic syndrome instead [3-5].

Meanwhile, *the best available evidence *from three consecutive large meta-analyses systematically had shown that people with metabolic syndrome are at increased risk of cardiovascular events [6-8]. The most recent and largest of them [8] included near one million patients (total n = 951,083). The investigators concluded that the metabolic syndrome is associated with a 2-fold increase in cardiovascular outcomes and a 1.5-fold increase in all-cause mortality rates. The meta-analysis showed that the point estimates for cardiovascular risk were consistently higher in women vs. men. A very important finding of this study demonstrated that cardiovascular risk was still high in patients with the metabolic syndrome but without diabetes.

The prognostic importance of the metabolic syndrome, compared with that of the sum of its individual components has repeatedly been challenged [5,9,10]. For example, in a cohort study of 2,815 patients [9], the risk of cardiovascular disease (CVD) mortality associated with the metabolic syndrome (HR: 2.53; 95% CI: 1.74 to 3.67) was similar to the risk associated with impaired fasting glucose (HR: 2.87; 95% CI: 1.96 to 4.20). However, it seems that most of the published reports indicate that the syndrome predicts cardiovascular events or/and diabetes independently from other conventional risk factors [11-16]. Our group has shown that metabolic syndrome is a strong independent predictor of mortality and morbidity in patients with acute coronary syndrome [16]. It should be specifically pointed out that patients with hyperglycemia and metabolic syndrome had higher mortality rates compared with patients with the same hyperglycemia but without metabolic syndrome (for example 30-day mortality rates respectively 8.3% vs. 2.5%, p <0.05)!

This situation resembled the old and long- lasting discussion regarding clinical significance of insulin resistance: over the past years it has been recognized that insulin resistance is an independent risk factor for the development of type 2 diabetes mellitus, whereas its association with major cardiovascular events remained controversial. We have previously demonstrated the independent association of insulin resistance with major cardiovascular events and mortality [17]. After multivariable adjustments, measurable effect of insulin resistance was somewhat attenuated but remained significant. Similarly, insulin resistance was associated with >2-fold increased age-adjusted risk for the development of diabetes in nondiabetic subjects. After adjustment for multiple potential confounders, the prediction conferred by the insulin resistance for new diabetes was substantially attenuated, mainly after inclusion of body mass index in the model, remaining yet strongly significant. Consequently, insulin resistance and body mass index are most likely associated with diabetes development by partially (but not completely) reciprocated mechanisms.

One of the ways to hit the metabolic syndrome is an utterly simplistic view on this concept as a predictive tool only. Of course, the presence of the metabolic syndrome possesses a definite predictive value, but first of all it is a widely accepted concept regarding a biological condition based on the complex and interrelated pathophysiological mechanisms starting from excess central adiposity and insulin resistance. Therefore, it is completely unfair to compare it with statistically constructed predictive tools, including stronger prognostic variables even unrelated to each other from the biological point of view. For example, in the criteria for metabolic syndrome (in contrast to Framingham score) age and cholesterol (presumably LDL-C) levels are not included, as well as a variety of strong predictors used in other risk-stratification scores: previous myocardial infarction, heart failure, smoking, family history, etc. However, the metabolic syndrome identifies additional important residual vascular risk mainly associated with insulin resistance and atherogenic dyslipidemia (low HDL-C, high triglycerides, small dense LDL-C). Therefore, the metabolic syndrome could be a useful additional contributor in estimation of global cardiovascular risk beyond age, high LDL-C or other standard risk factors.

Moreover, even critics of the metabolic syndrome concept should agree that obesity, dysglycemia, dyslipidemia and hypertension coexist more frequently than predicted by chance. These common chronic conditions (and components of the metabolic syndrome) have partially *overlapping *mechanisms of pathogenic actions mediated through common metabolic pathways. Therefore their total combined effect could be less than the summed of the individual effects.

People with the metabolic syndrome usually pass through the phases of excessive adipogenesis (obesity), nuclear peroxisome proliferator-activated (PPAR) receptors modulation, insulin resistance, hyperinsulinemia, impaired glucose postprandial and fasting levels [2,18-22]. Fasting glucose is presumed to remain normal or borderline as long as insulin hypersecretion can compensate for insulin resistance. The fall in insulin secretion (due to pancreatic beta cells stress and damage) leading to hyperglycemia occurs as a late phenomenon and, in fact, separates the patients with metabolic syndrome from those with or without overt diabetes.

Development of insulin resistance in consequence of excess central adiposity has been considered to be key event in the origin and progression of the metabolic syndrome. It represents a complex interaction of maladaptive characteristics related to impaired insulin action at target organs and external factors such as genetics and environment. It is likely that the molecular factors that underlie insulin resistance (mediated in part via nuclear PPAR) contribute for many of the clinical components of the metabolic syndrome, although the precise associations remain still weakly understood [2,21,23-26].

Emerging multiple areas of metabolic syndrome research interests include nowadays heterogeneous topics like as adiponectin, angiotensinogen, resistin, and leptin secretion [27-29], nonalcoholic fatty liver disease and liver steatosis [30-32], hyperuricemia [33], genetic predisposition [34,35]; the role of inflammation, interleukins and high-sensitivity C-reactive protein [36-39]; age and gender specific profiles [40,41], the possible ways for treatment optimization [42-47] and several additional matters.

The concept that the metabolic syndrome is a consequence of obesity and insulin resistance, provides a useful "life-style changes" approach for prevention and treatment: caloric restriction, weight-loss and increased physical activity. The next step could theoretically be pharmacological interventions such as metformin, acarbose, fibrates, weight-loss drugs (currently only orlistat is practically available) and perhaps glucagon-like peptide-1 agonists [48-52]. A third step should probably be kept for bariatric surgery [53,54].

In conclusion, currently available evidences strongly support the evolving concept of the metabolic syndrome as an important clustering of the cardiovascular risk factors and diabetes. Resembling Mark Twain's renowned saying [55], the reports of its death are an exaggeration. The recognition, prevention, and treatment of the metabolic syndrome and its underlying risk factors should become an important approach for the reduction of cardiovascular disease burden in the general population. However, future problem-oriented research is needed to improve and unify the diagnostic criteria for the syndrome, its genetic and environmental basis and the optimal medical management.

## Abbreviations

CVD: cardiovascular disease; LDL-C: low density lipoprotein - cholesterol, HDL-C:high density lipoprotein-cholesterol; PPAR: peroxisome proliferator-activated receptor.

## Competing interests

The authors declare that they have no competing interests.

## Authors' contributions

Both authors have equally contributed in the conception and drafting of the manuscript and read and approved its final version.

## References

[B1] Alberti KG Eckel RH Grundy SM Zimmet PZ Cleeman JI Donato KA Fruchart JC James WP Loria CM Smith SC Jr International Diabetes Federation Task Force on Epidemiology and Prevention Hational Heart Lung Blood Institute; American Heart Association World Heart Federation International Atherosclerosis Society International Association for the Study of Obesity Harmonizing the metabolic syndrome: a joint interim statement of the International Diabetes Federation Task Force on Epidemiology and Prevention; National Heart, Lung, and Blood Institute; American Heart Association; World Heart Federation; International Atherosclerosis Society; and International Association for the Study of Obesity Circulation 2009 120 1640 1645 10.1161/CIRCULATIONAHA.109.192644 19805654

[B2] Eckel RH Alberti KG Grundy SM Zimmet PZ The metabolic syndrome Lancet 2010 375 181 183 10.1016/S0140-6736(09)61794-3 20109902

[B3] Kahn R Buse J Ferrannini E Stern M The metabolic syndrome: time for a critical appraisal. Joint statement from the American Diabetes Association and the European Association for the Study of Diabetes Diabetologia 2005 48 1684 1699 10.1007/s00125-005-1876-2 16079964

[B4] Kahn R Metabolic syndrome--what is the clinical usefulness? Lancet 2008 371 1892 1893 10.1016/S0140-6736(08)60731-X 18501420

[B5] Sattar N McConnachie A Shaper AG Blauw GJ Buckley BM de Craen AJ Ford I Forouhi NG Freeman DJ Jukema JW Lennon L Macfarlane PW Murphy MB Packard CJ Stott DJ Westendorp RG Whincup PH Shepherd J Wannamethee SG Can metabolic syndrome usefully predict cardiovascular disease and diabetes? Outcome data from two prospective studies Lancet 2008 371 1927 1935 10.1016/S0140-6736(08)60602-9 18501419

[B6] Gami AS Witt BJ Howard DE Erwin PJ Gami LA Somers VK Montori VM Metabolic syndrome and risk of incident cardiovascular events and death: a systematic review and meta-analysis of longitudinal studies J Am Coll Cardiol 2007 49 403 414 10.1016/j.jacc.2006.09.032 17258085

[B7] Galassi A Reynolds K He J Metabolic syndrome and risk of cardiovascular disease: a meta-analysis Am J Med 2006 119 812 819 10.1016/j.amjmed.2006.02.031 17000207

[B8] Mottillo S Filion KB Genest J Joseph L Pilote L Poirier P Rinfret S Schiffrin EL Eisenberg MJ The metabolic syndrome and cardiovascular risk. A systematic review and meta-analysis J Am Coll Cardiol 2010 56 1113 1132 10.1016/j.jacc.2010.05.034 20863953

[B9] Hunt KJ Resendez RG Williams K Haffner SM Stern MP San Antonio Heart Study National Cholesterol Education Program versus World Health Organization metabolic syndrome in relation to all-cause and cardiovascular mortality in the San Antonio Heart Study Circulation 2004 110 1251 1257 10.1161/01.CIR.0000140762.04598.F9 15326061

[B10] Haring R Wallaschofski H Nauck M Felix SB Schmidt CO Dörr M Sauer S Wilmking G Völzke H Total and Cardiovascular Disease Mortality Predicted by Metabolic Syndrome is Inferior Relative to its Components Exp Clin Endocrinol Diabetes 2010 118 685 691 10.1055/s-0030-1261876 20625974

[B11] Sundstrom J Riserus U Byberg L Zethelius B Lithell H Lind L Clinical value of the metabolic syndrome for long term prediction of total and cardiovascular mortality: prospective, population based cohort study BMJ 2006 332 878 82 10.1136/bmj.38766.624097.1F 16510492 PMC1440609

[B12] Simons LA Simons J Friedlander Y McCallum J Does a diagnosis of the metabolic syndrome provide additional prediction of cardiovascular disease and total mortality in the elderly? The Dubbo Study Med J Aust 2007 186 400 403 17437393 10.5694/j.1326-5377.2007.tb00972.x

[B13] Guzder RN Gatling W Mullee MA Byrne CD Impact of metabolic syndrome criteria on cardiovascular disease risk in people with newly diagnosed type 2 diabetes Diabetologia 2006 49 49 55 10.1007/s00125-005-0063-9 16341841

[B14] Kasai T Miyauchi K Kurata T Okazaki S Kajimoto K Kubota N Daida H Impact of metabolic syndrome among patients with and without diabetes mellitus on long-term outcomes after percutaneous coronary intervention Hypertens Res 2008 31 235 241 10.1291/hypres.31.235 18360042

[B15] Dohi T Miyauchi K Kasai T Kajimoto K Kubota N Tamura H Yokoyama T Kojima T Yokoyama K Kurata T Daida H Impact of metabolic syndrome on 10-year clinical outcomes among patients with acute coronary syndrome Circ J 2009 73 1454 1458 10.1253/circj.CJ-08-1122 19531901

[B16] Feinberg MS Schwartz R Tanne D Fisman EZ Hod H Zahger D Schwammethal E Eldar M Behar S Tenenbaum A Impact of the metabolic syndrome on the clinical outcomes of non-clinically diagnosed diabetic patients with acute coronary syndrome Am J Cardiol 2007 99 667 672 10.1016/j.amjcard.2006.10.023 17317369

[B17] Tenenbaum A Adler Y Boyko V Tenenbaum H Fisman EZ Tanne D Lapidot M Schwammenthal E Feinberg MS Matas Z Motro M Behar S Insulin resistance is associated with increased risk of major cardiovascular events in patients with preexisting coronary artery disease Am Heart J 2007 153 559 565 10.1016/j.ahj.2007.01.008 17383294

[B18] Tenenbaum A Motro M Schwammenthal E Fisman EZ Macrovascular complications of metabolic syndrome: an early intervention is imperative Int J Cardiol 2004 97 167 172 10.1016/j.ijcard.2003.07.033 15458679

[B19] Hayden MR Tyagi SC Intimal redox stress: accelerated atherosclerosis in metabolic syndrome and type 2 diabetes mellitus. Atheroscleropathy Cardiovasc Diabetol 2002 1 3 10.1186/1475-2840-1-3 12392600 PMC140143

[B20] Porte D Jr Kahn SE Beta-cell dysfunction and failure in type 2 diabetes: potential mechanisms Diabetes 2001 50 Suppl. 1 S160 3 10.2337/diabetes.50.2007.S160 11272181

[B21] Tenenbaum A Fisman EZ Motro M Metabolic syndrome and type 2 diabetes mellitus: focus on peroxisome proliferator activated receptors. (PPAR) Cardiovasc Diabetol 2003 2 4 10.1186/1475-2840-2-4 12834541 PMC153546

[B22] Tenenbaum H Behar S Boyko V Adler Y Fisman EZ Tanne D Lapidot M Schwammenthal E Feinberg M Matas Z Motro M Tenenbaum A Long-term effect of bezafibrate on pancreatic beta-cell function and insulin resistance in patients with diabetes Atherosclerosis 2007 194 265 271 10.1016/j.atherosclerosis.2006.08.005 16970952

[B23] Nesto RW Correlation between cardiovascular disease and diabetes mellitus: current concepts Am J Med 2004 116 Suppl 5A 11S 22S 10.1016/j.amjmed.2003.10.016 15019859

[B24] Reaven GM Role of insulin resistance in human disease (syndrome X): an expanded definition Annu Rev Med 1993 44 121 131 10.1146/annurev.me.44.020193.001005 8476236

[B25] Jadhav S Petrie J Ferrell W Insulin resistance as a contributor to myocardial ischaemia independent of obstructive coronary atheroma: a role for insulin sensitisation? Heart 2004 90 1379 13 83 10.1136/hrt.2004.035170 15547007 PMC1768561

[B26] Cleland SJ Petrie JR Small M Elliott HL Connell JM Insulin action is associated with endothelial function in hypertension and type 2 diabetes Hypertension 2000 35 507 511 10642350 10.1161/01.hyp.35.1.507

[B27] Comuzzie AG Tejero ME Funahashi T Martin LJ Kissebah A Takahashi M Kihara S Tanaka S Rainwater DL Matsuzawa Y MacCluer JW Blangero J The genes influencing adiponectin levels also influence risk factors for metabolic syndrome and type 2 diabetes Hum Biol 2007 79 191 200 10.1353/hub.2007.0029 18027814

[B28] Hiuge A Tenenbaum A Maeda N Benderly M Kumada M Fisman EZ Tanne D Matas Z Hibuse T Fujita K Nishizawa H Adler Y Motro M Kihara S Shimomura I Behar S Funahashi T Effects of peroxisome proliferator-activated receptor ligands, bezafibrate and fenofibrate, on adiponectin level Arterioscler Thromb Vasc Biol 2007 27 635 641 10.1161/01.ATV.0000256469.06782.d5 17194889

[B29] Cornier MA Dabelea D Hernandez TL Lindstrom RC Steig AJ Stob NR Van Pelt RE Wang H Eckel RH The metabolic syndrome Endocr Rev 2008 29 777 822 10.1210/er.2008-0024 18971485 PMC5393149

[B30] Steinvil A Shapira I Ben-Bassat OK Cohen M Vered Y Berliner S Rogowski O The association of higher levels of within-normal-limits liver enzymes and the prevalence of the metabolic syndrome Cardiovasc Diabetol 2010 9 30 10.1186/1475-2840-9-30 20633271 PMC2915953

[B31] Tilg H Moschen A Update on nonalcoholic fatty liver disease: genes involved in nonalcoholic fatty liver disease and associated inflammation Curr Opin Clin Nutr Metab Care 2010 13 391 396 10.1097/MCO.0b013e32833a87cc 20473151

[B32] Kawamoto R Tabara Y Kohara K Miki T Kusunoki T Takayama S Abe M Katoh T Ohtsuka N High-sensitivity c-reactive protein and gamma-glutamyl transferase levels are synergistically associated with metabolic syndrome in community-dwelling persons Cardiovasc Diabetol 2010 9 87 10.1186/1475-2840-9-87 21143879 PMC3014885

[B33] Borges RL Ribeiro AB Zanella MT Batista MC Uric acid as a factor in the metabolic syndrome Curr Hypertens Rep 2010 12 113 119 10.1007/s11906-010-0098-2 20424936

[B34] Junyent M Arnett DK Tsai MY Kabagambe EK Straka RJ Province M An P Lai CQ Parnell LD Shen J Lee YC Borecki I Ordovás JM Genetic variants at the PDZ-interacting domain of the scavenger receptor class B type I interact with diet to influence the risk of metabolic syndrome in obese men and women J Nutr 2009 139 842 848 10.3945/jn.108.101196 19321583 PMC2714388

[B35] Garaulet M Madrid JA Chronobiology, genetics and metabolic syndrome Curr Opin Lipidol 2009 20 127 134 10.1097/MOL.0b013e3283292399 19276891

[B36] Trøseid M Seljeflot I Arnesen H The role of interleukin-18 in the metabolic syndrome Cardiovasc Diabetol 2010 9 11 20331890 10.1186/1475-2840-9-11 PMC2858122

[B37] Kong AP Chan NN Chan JC The role of adipocytokines and neurohormonal dysregulation in metabolic syndrome Curr Diabetes Rev 2006 2 397 407 18220644 10.2174/1573399810602040397

[B38] Fisman EZ Tenenbaum A The ubiquitous interleukin-6: a time for reappraisal Cardiovasc Diabetol 2010 9 62 10.1186/1475-2840-9-62 20937099 PMC2959009

[B39] Calabro P Yeh ET Intra-abdominal adiposity, inflammation, and cardiovascular risk: new insight into global cardiometabolic risk Curr Hypertens Rep 2008 10 32 38 10.1007/s11906-008-0008-z 18367024

[B40] Regitz-Zagrosek V Lehmkuhl E Mahmoodzadeh S Gender aspects of the role of the metabolic syndrome as a risk factor for cardiovascular disease Gend Med 2007 4 Suppl B S162 177 10.1016/S1550-8579(07)80056-8 18156101

[B41] Moebus S Balijepalli C Lösch C Göres L von Stritzky B Bramlage P Wasem J Jöckel KH Age- and sex-specific prevalence and ten-year risk for cardiovascular disease of all 16 risk factor combinations of the metabolic syndrome - A cross-sectional study Cardiovasc Diabetol 2010 9 34 10.1186/1475-2840-9-34 20696055 PMC2929217

[B42] Tenenbaum A Motro M Fisman EZ Tanne Boyko V Behar S Bezafibrate for the secondary prevention of myocardial infarction in patients with metabolic syndrome Arch Intern Med 2005 165 1154 1160 10.1001/archinte.165.10.1154 15911729

[B43] Tenkanen L Manttari M Kovanen PT Virkkunen H Manninen V Gemfibrozil in the treatment of dyslipidemia: an 18-year mortality follow-up of the Helsinki Heart Study Arch Intern Med 2006 166 743 748 10.1001/archinte.166.7.743 16606810

[B44] Zidek W Naditch-Brûlé L Perlini S Farsang C Kjeldsen SE Blood pressure control and components of the metabolic syndrome: the GOOD survey Cardiovasc Diabetol 2009 8 51 10.1186/1475-2840-8-51 19754934 PMC2757016

[B45] Tenenbaum A Fisman EZ Boyko V Benderly M Tanne D Haim M Matas Z Motro M Behar S Attenuation of progression of insulin resistance in patients with coronary artery disease by bezafibrate Arch Intern Med 2006 166 737 741 10.1001/archinte.166.7.737 16606809

[B46] Tenenbaum A Motro M Fisman EZ Adler Y Shemesh J Tanne D Leor J Boyko V Schwammenthal E Behar S Effect of bezafibrate on incidence of type 2 diabetes mellitus in obese patients Eur Heart J 2005 26 2032 2038 10.1093/eurheartj/ehi310 15872029

[B47] Hansen BC Tigno XT Benardeau A Meyer M Sebokova E Mizrahi J Effects of aleglitazar, a balanced dual peroxisome proliferator-activated receptor alpha/gamma agonist on glycemic and lipid parameters in a primate model of the metabolic syndrome Cardiovasc Diabetol 2011 10 7 10.1186/1475-2840-10-7 21251281 PMC3037308

[B48] Sullivan SD Ratner RE Should the Metabolic Syndrome Patient with Prediabetes Be Offered Pharmacotherapy? Curr Diab Rep 2011 in press 21207204 10.1007/s11892-010-0170-y

[B49] Tenenbaum A Motro M Fisman EZ Schwammenthal E Adler Y Goldenberg I Leor J Boyko V Mandelzweig L Behar S Peroxisome proliferator-activated receptors ligand bezafibrate for prevention of type 2 diabetes mellitus in patients with coronary artery disease Circulation 2004 109 2197 2202 10.1161/01.CIR.0000126824.12785.B6 15123532

[B50] Lukacova-Zib I Gopalakrishnan G Therapeutic options for the prevention of type 2 diabetes mellitus in the metabolic syndrome Mt Sinai J Med 2010 77 524 532 10.1002/msj.20204 20960554

[B51] Tenenbaum A Fisman EZ "If it ain't broke, don't fix it": a commentary on the positive-negative results of the ACCORD Lipid study Cardiovasc Diabetol 2010 9 24 10.1186/1475-2840-9-24 20550659 PMC2893121

[B52] Scott R O'Brien R Fulcher G Pardy C D'Emden M Tse D Taskinen MR Ehnholm C Keech A Fenofibrate Intervention and Event Lowering in Diabetes (FIELD) Study Investigators Effects of fenofibrate treatment on cardiovascular disease risk in 9,795 individuals with type 2 diabetes and various components of the metabolic syndrome: the Fenofibrate Intervention and Event Lowering in Diabetes (FIELD) study Diabetes Care 2009 32 493 498 10.2337/dc08-1543 18984774 PMC2646035

[B53] Pontiroli AE Laneri M Veronelli A Frigè F Micheletto G Folli F Adami G Scopinaro N Biliary pancreatic diversion and laparoscopic adjustable gastric banding in morbid obesity: their long-term effects on metabolic syndrome and on cardiovascular parameters Cardiovasc Diabetol 2009 8 37 10.1186/1475-2840-8-37 19619292 PMC3224750

[B54] Lahsen R Berry M Surgical interventions to correct metabolic disorders Br J Diabetes Vasc Dis 2010 10 143 147 10.1177/1474651410370302

[B55] Powers R Mark Twain: A Life 2005 New York, NY: Free Press

